# Editorial: Mindful and intuitive eating: insights and interventions

**DOI:** 10.3389/fnut.2026.1837530

**Published:** 2026-04-13

**Authors:** Costela Lacrimioara Serban, Monica Tarcea, Sorin Ursoniu

**Affiliations:** 1 Department of Functional Sciences, Discipline of Public Health, Center for Translational Research and Systems Medicine, “Victor Babes” University of Medicine and Pharmacy, Timisoara, Romania; 2 Department of Community Nutrition and Food Safety, Faculty of Medicine, George Emil (G.E.) Palade University of Medicine, Pharmacy, Science and Technology From Târgu Mureş, Târgu Mureş, Romania

**Keywords:** eating behaviors, emotional eating, Intuitive eating, mindful eating, prevention of chronic diseases, psychological health

The way people eat is increasingly recognized as being just as important as what they eat. In recent years, mindful and intuitive eating has emerged as an influential framework in nutrition, psychology, and public health, reflecting a broader shift from narrow, nutrient-centered or weight-centered models of eating behavior toward a broader understanding of eating as something influenced by body, emotions, and the social environment (1). In a context marked by rising rates of obesity, chronic disease, psychological distress, and disordered eating, these frameworks offer valuable perspectives for understanding not only what people eat, but also how, why, and under what circumstances eating occurs (2–4).

The contributions gathered in this Research Topic highlight both the diversity of this field and its increasing conceptual and methodological maturity. Together, they show that mindful and intuitive eating are not isolated concepts, but part of a broader scientific effort to understand eating behavior through multiple and complementary lenses, including: motivation, interoception, affective vulnerability, sensory pleasure, and therapeutic application. Importantly, the articles in this Research Topic also illustrate the interdisciplinary nature of this research area, bringing together psychometrics, nutritional science, clinical psychology, neuroscience, and translational thinking.

One of the strengths of this Research Topic is its attention to measurement and the cultural context. In their cross-cultural adaptation and linguistic validation of the Eating Motivation Survey among older adults in China, Wu et al. underline the importance of ensuring that eating-related constructs are meaningfully translated across languages, age groups, and cultural settings. Their work shows that motivations for eating are embedded in everyday realities and social contexts and that robust tools are essential for both research and intervention design, particularly in populations such as older adults, where healthy aging is increasingly relevant.

Cultural specificity is also central to the study by Albajri and Naseeb, who assessed intuitive eating in a Saudi Arabian population using an Arabic version of the Intuitive Eating Scale-2. Their findings indicate that intuitive eating is associated with several sociodemographic and nutritional factors, including sex, age, body mass index, education, employment status, and recent weight change. Beyond expanding the evidence base geographically, this article reminds us that intuitive eating cannot be fully understood outside of the social and cultural environments in which eating behaviors are formed and maintained.

At the same time, the Research Topic avoids uncritically idealizing healthy eating concepts. The study by Sanlier et al. explores the associations between healthy eating obsession, clinical eating disorder impairment, and health anxiety, drawing attention to the potential dark side of the pursuit of health. This contribution is particularly important because it complicates overly simplistic narratives about “eating well,” showing that a strong focus on healthy eating can, in some individuals, become rigid, anxiety-laden, and impairing. Such findings reinforce the need for nuance in both research and public health messaging.

The psychological dimension of eating behavior is further developed in the article by Schamarek et al., which examines the relationship between depressive symptoms, body mass index, and trait eating behavior in a non-clinical sample. By identifying trait disinhibition as a mediator of the association between depressive symptoms and BMI, the authors offer an important insight into the behavioral pathways through which emotional distress may shape eating-related outcomes. This study strengthens the argument that eating behavior research must engage more directly with mental health, even outside formal clinical populations.

This topic also broadens the discussion beyond self-report measures and psychopathology by including sensory and neurobehavioral perspectives. In their study on the pleasantness and neural activity induced by the multimodal crispiness of monaka ice cream, Yamamoto et al. contribute an original perspective on the embodied and hedonic dimensions of eating. Although it differs somewhat from traditional mindful or intuitive eating research, this work highlights an essential point: food-related experience is not only cognitive or behavioral but also sensory, affective, and deeply rooted in reward processing. It reminds us that pleasure is not peripheral to eating behavior; it is central to it.

Finally, Fernandez-Demeneghi et al. propose mindful eating as a therapeutic frontier in nutritional psychiatry, offering a conceptual bridge between eating behavior, emotional regulation, and metabolic health. Their perspective moves beyond reductionist nutritional models focused solely on nutrients and dietary prescriptions, instead emphasizing attention, awareness, interoception, reward regulation, stress responsiveness, and the gut-brain axis. In doing so, the article advances an important translational vision for the field and suggests that mindful eating may be relevant not only to dietary behavior but also to broader psychological and psychiatric wellbeing.

Taken together, the articles included in this Research Topic suggest that mindful and intuitive eating should be understood not simply as intervention labels but as entry points into a broader science of eating behavior. They encourage us to consider how people's relationships with food are shaped by culture, aging, body weight, mood, anxiety, sensory experience, and meaning-making. They also point to future research priorities, including stronger conceptual integration, improved measurement, longitudinal designs, and the development of culturally sensitive and clinically relevant interventions.

As this field continues to evolve, caution remains necessary. Not all proposed frameworks apply equally across populations, and not all promising concepts are yet supported by intervention-level evidence. However, the contributions collected here clearly indicate that research on mindful and intuitive eating is moving in a productive direction: away from rigid, prescriptive, and one-dimensional understandings of food behavior and toward a more nuanced view of eating as a lived, relational, and context-dependent human experience.

Mindful eating and intuitive eating represent innovative, non-diet approaches to fostering healthier eating behaviors, with growing evidence linking them to reductions in binge eating and emotional eating, as well as greater weight management success. Multi-component interventions appear most effective for disordered eating but may not surpass alternatives, with effects varying by individual traits and context. Baseline mindful eating also predicts long-term weight outcomes, underscoring its role in sustained interventions (5).

The articles and reviews in this Research Topic advance the field by dissecting mechanisms, intervention efficacy, and clinical applications. As obesity and eating disorders rise, integrating mindful and intuitive eating into medical practice offers a patient-centered path forward, warranting further mechanistic and comparative research to refine protocols.

These practices emphasize heightened awareness of eating experiences, bodily sensations, and internal cues such as fullness, in contrast to traditional restrictive dieting.

We hope that this Research Topic stimulates further interdisciplinary dialogue and inspires new work that can refine theory, strengthen evidence, and inform more compassionate and person-centered approaches to nutrition and health.
